# Expression Profiling of Circular RNAs in Early Pregnant Jianghuai Buffaloes

**DOI:** 10.3390/ani12202748

**Published:** 2022-10-13

**Authors:** Qiuchen Liu, Mengya Zhang, Tenglong Guo, Sucheng Wu, Yanfeng Zong, Changzhi Xu, Zhihua Zhu, Yunhai Zhang, Zubing Cao

**Affiliations:** 1 Anhui Province Key Laboratory of Local Livestock and Poultry, Genetical Resource Conservation and Breeding, College of Animal Science and Technology, Anhui Agricultural University, Hefei 230036, China; 2 Department of Animal Engineering, Huaian Bioengineering Vocational College, Huaian 223001, China

**Keywords:** buffalo, gestation establishment, circRNA, reproductive efficiency

## Abstract

**Simple Summary:**

CircRNA transcriptome sequencing technology is widely used in the study of germ-line stem cell proliferation and differentiation and early embryonic development, but the research on early pregnancy diagnosis in female animals is still in the preliminary stage, and the specific regulatory mechanism has not been reported. Here, we identified numerous differentially expressed circRNAs (DECs) in the venous blood of the distinctive local breed of Chinese Jianghuai buffalo. Analysis of the enrichment showed that DECs were mainly enriched in the epidermal growth factor receptor-signaling pathway that is important for embryonic development and pregnancy maintenance. These findings have provided a better understanding of buffalo pregnancy establishment and a potential basis for improving early pregnancy diagnosis techniques in buffalo.

**Abstract:**

Circular RNA (circRNA) is expressed in cells and tissues of several species. However, the expression of circRNAs in the blood of Jianghuai buffaloes during early pregnancy has not been reported. In this study, we identified the DECs in the blood of Jianghuai buffaloes and annotated the functions of these DECs. The results showed that there were 890 DECs between the pregnant and non-pregnant groups, of which more than 80% were exon-derived circRNAs, including 323 up-regulated circRNAs and 567 down-regulated circRNAs. Enrichment analysis revealed that DECs were mainly enriched in the epidermal growth factor receptor-signaling pathway important for embryonic development and pregnancy maintenance. In addition, most DECs have multiple miRNA targets, suggesting that these DECs have the potential to function as miRNA sponges. In conclusion, several DECs are present between pregnant and non-pregnant Jianghuai buffaloes, and these DECs are associated with embryo implantation and pregnancy establishment.

## 1. Introduction

The formation of a mammalian pregnancy is an evolutionarily conserved and complex biological process that begins with the union of sperm and oocyte in the fallopian tube and proceeds through a series of oogeneses into the uterine cavity in a floating form [1], where the free-floating embryo releases signals in the uterus to maintain progesterone production, thereby maintaining and continuing the pregnancy [2,3]. Early pregnancy diagnosis is one way to improve female animals’ reproductive efficiency. With the rapid development of large-scale and intensive cattle breeding [4,5,6], there is an urgent need for more accurate, efficient, and convenient early pregnancy diagnosis methods to shorten the calving interval of buffaloes, reduce the feeding costs of empty pregnant buffaloes and improve reproductive efficiency [7,8]. The Jianghuai buffalo is a marsh buffalo mainly distributed to the south of the Huaihe River and north of the Yangtze River. Its coat’s fur is mainly green-grey, and there is no white spot or seasonal black spot in the whole herd. The Jianghuai buffalo has a large body, strong trunk, and is well structured. The oestrus cycle of Jianghuai buffalo is about 21 ± 2 days, and the gestation period is about 308 ± 4 d. It has been certified by the China Livestock and Poultry Genetic Resources Committee and is a good local breed with a long history in the Anhui Province. It is docile in temperament, good at walking in the mire, resistant to rough feeding and has strong in-service performance. However, in recent years, the population of Jianghuai buffalo has shown a downward trend. At present, the population of Jianghuai buffalo is only about 6120, among which there are about 3000 cows with reproductive ability. One of the main reasons for the sharp decline in the population of Jianghuai buffalo is the long calving interval of cows and the low survival rate of calves, which leads to overall low reproductive efficiency [9,10]. Early pregnancy diagnosis after breeding can effectively shorten the calving interval and improve the overall fertility of the cow [11,12], thus increasing the number and quality of the herd [13]. As modern herd management moves towards a high level of development, cattle breeding techniques are being developed to improve overall herd fertility, and early pregnancy diagnosis methods currently used in the cattle industry include rectal palpation [14,15], ultrasound, and immunological diagnosis through the detection of pregnancy-related biomarkers [16]. However, all of these early pregnancy diagnostic methods have certain drawbacks that make them difficult to meet the needs of the current cattle industry [10].

CircRNA is a covalently closed circRNA molecule that can be stably present in a variety of eukaryotic cells [17,18,19]. In recent years, with the development of high-throughput sequencing technology and bio-information technology, circRNA has become a current research hotspot, and some mysteries about circRNA have been gradually unraveled [20,21]. According to the source of circRNA sequences, circRNAs can be divided into four categories: the first type is circRNAs that all originate from exons of the host gene (Exonic circRNA, EciRNA); the second type is lasso-type circRNAs that all originate from introns (Circular intronic RNA, ciRNA); the third type is (Exon-intron circRNA, EIciRNA); the fourth type includes antisense circRNA (Antisense circular RNA) derived from antisense strand transcripts, circRNA derived from intergenic sequences, or other unannotated genomic sequences (Intergenic circular). The vast majority of circRNAs belong to exon-type circRNAs, which are also more conserved than other types of circRNAs. Currently, thousands of circRNAs have been identified in the peripheral blood of normal humans, many of which are more highly expressed than the mRNAs of their source genes and therefore more easily detected [17]. Also, the unique circular structure of circRNAs, as opposed to linear RNAs, makes them resistant to digestion by nucleic acid exonucleases or ribonucleases [18,20,22]. Therefore, these stably present and highly expressed circRNAs can be used as molecular markers for non-invasive liquid biopsies or non-invasive diagnostic methods [23]. In addition, some studies have found that circRNAs are closely associated with processes such as mammalian pregnancy recognition and are expected to be used as a novel early pregnancy diagnostic method in animal husbandry [23,24].

Here, we examined blood samples from day 25 of gestation, and a large number of differential circRNAs were present in blood samples from day 25 of gestation compared to non-pregnant cows. We hypothesized that the pregnancy status of buffalo may be related to circRNA characteristics in blood. This study aimed to determine the differences in circRNA expression patterns in the blood of pre- and post-pregnant cows at day 25 of gestation.

## 2. Materials and Methods

### 2.1. Blood Sample Collection

A total of 20 cows were included in the study at a Jianghuai buffalo farm in Dingyuan County, Chuzhou City, Anhui Province, China. The cows used in this study were fed continuously under the same rearing conditions for at least 5 months. The cows were 3–5 years old, in good health, of similar body weight, with moderate body mass, and no history of reproductive tract disease. Cows were given two injections of sodium D-chlorprostol (Yuanye, China) for simultaneous estrus, 4 mL each with an interval of 11 days. The cows were divided into two groups: one group underwent artificial insemination (AI) and the other group underwent no treatment after estrus. Three days later, frozen sperm with vigor greater than 0.7 after thawing were selected for insemination, and the date of AI was considered day 0. Blood samples were collected from both groups of cows on day 25 after AI. Gestation group status per cow was evaluated 60 days after AI using ultrasound technology. Blood samples from pregnant cows were used as the pregnant group (*n* = 3) and blood samples from cows without AI were used as the control group.

### 2.2. Construction and Sequencing of CircRNA Libraries

Separation and purification of total RNA was performed using Trizol reagent (Invitrogen, Carlsbad, CA, USA) according to the manufacturer’s protocol. The RNA quantity and purification of each sample was determined by quantifying with NanoDrop ND-1000. (NanoDrop, Wilmington, DE, USA). The RNA integrity for RIN values greater than 7.0 was assessed by Agilent 2100. Ribosomal RNA was removed using around 5 μg of total RNA according to the manual of the Ribo-Zero™ rRNA Removal Kit (Illumina, San Diego, CA, USA). The rest of the RNA was then treated with Rnase R (Epicentre Inc. Madison, WI, USA). The RNA segments were then reverse transcribed into cDNA, which was next utilized to synthesize U-labeled second-strand DNAs. The products were subsequently expanded by PCR. Finally, we conducted paired-end sequencing on an Illumina Hiseq 4000 (LC Bio, Hangzhou, China).

### 2.3. Bioinformatic Analysis of CircRNA

Cutadapt was used to purify the reads of the bases. FastQC was then applied to validate the quality of the sequences. We used Bowtie2 [25] and Tophat2 [26] for mapping the reads to the genome of Bubalus Bubalis (Ensemble database Index of https://ftp.ncbi.nlm.nih.gov/genomes/all/GCF/003/121/395/GCF_003121395.1_ASM312139v1/, accessed on 9 May 2019). Unmapped reads were remapped onto the genome with Tophat-fusion [27]. CIRCExplorer [28,29] and CIRI [30] were applied to assembling the mapped reads to circRNAs. All samples generated unique circRNAs. The DECs were selected with|log2 (fold change)| ≥ 1 and with statistical significance (*p*-adjust < 0.05) by *t*-test.

To analyze the function of host genes with DECs involved in common biological processes, host genes with DECs were selected for Gene Ontology (GO) analysis and Kyoto Encyclopaedia of Genes and Genomes (KEGG) analysis. This host information can be classified into 3 categories in the GO database: biological processes, cellular components, and molecular functions. KEGG database was used to analyze the biological mechanism and cellular pathway of host genes of DECs. (Established criteria: *p* adjusted <0.05), using GO and KEGG (Gene Ontology Resource (http://geneontology.org/, accessed on 20 July 2019) and KEGG: Kyoto Encyclopedia of Genes and Genomes (https://www.kegg.jp/kegg/,accessed on 23 July 2019)) enrichment analysis.

The post-transcriptional regulatory functions of miRNAs were predicted and analyzed using TargetScan (v7.0; targetscan.org (https://www.targetscan.org, accessed on 17 September 2019)) and Miranda (microRNA.org (https://link.zhihu.com/?target=http%3A//www.microRNA.org, accessed on 17 September 2019)).

### 2.4. Real-Time Quantitative Polymerase Chain Reaction (qPCR)

To identify the reliability of RNA-seq, five circRNAs that might be associated with pregnancy establishment were selected for qPCR, and the results were compared with RNA-seq data.

TRNzol Universal Reagent kit was used to sample total RNA extraction; Ribonuclease R (RNase R) was applied to remove linear RNA, and the components were added to an ice box, metal bath at 37 °C, 5 min; EasyScript One-Step gDNA Removal and cDNA Synthesis SuperMix kit for RNA reverse transcription on the ice boxe, reaction conditions: phase I, 25 °C, 10 min, phase II, 42 °C, 15 min, phase III, 85 °C, 5 s. Glyceraldehyde-3-phosphate dehydrogenase (*GAPDH*) was used as the internal reference, and qPCR analysis was performed using AceQ qPCR SYBR Master Mix and AB StepOnePlus. Reaction systems were prepared in PCR tubes using 3 replicate wells as technical replicates, and the systems were treated appropriately. The primers used in this study are listed in Supplementary Table S1.

### 2.5. Statistical Analysis

Three biological replicates were conducted for all experiments. Data were subjected to *t*-test or one way ANOVA (SPSS V17.0) and represented by mean ± standard error of magnitude (mean ± S.E.M.). *p* < 0.05 was deemed as statistically significant.

## 3. Results

### 3.1. Characteristics of CircRNAs Expressed in the Blood of Jianghuai Buffalo

**Figure 1:**
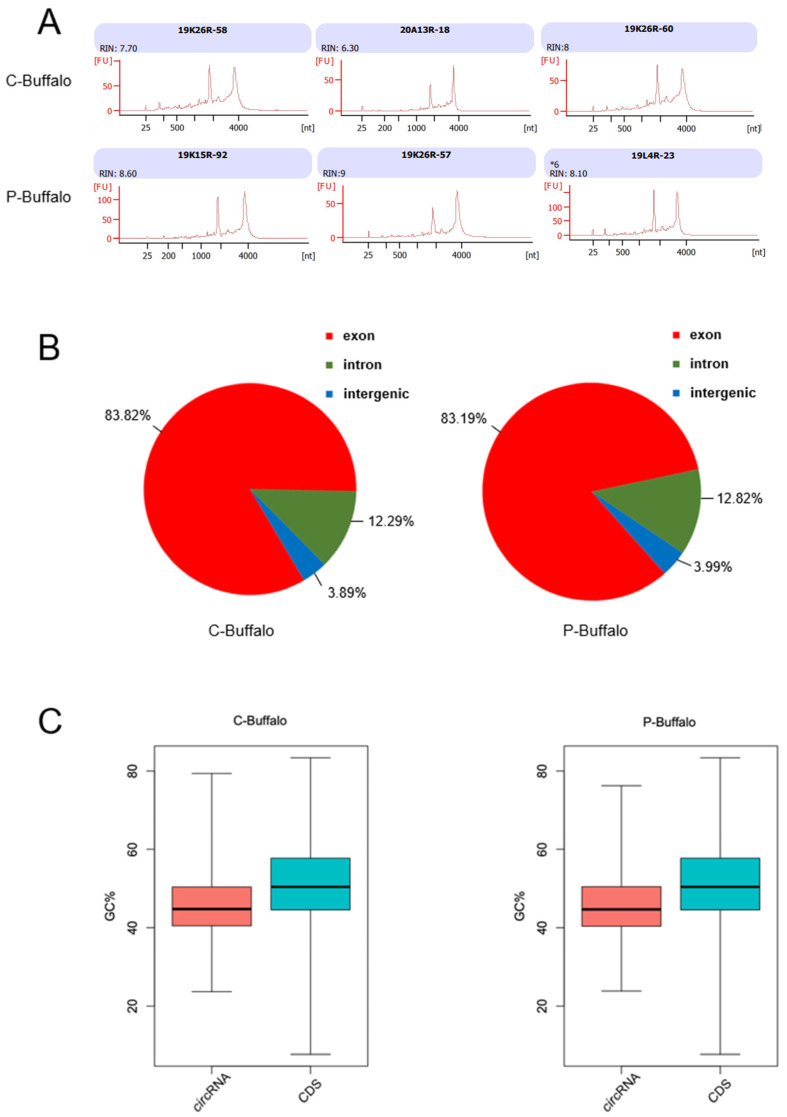
Characteristics of circRNAs expressed in the blood of Jianghuai buffalo. (**A**) Detection results of RNA quality in blood samples of Jianghuai buffalo. Total RNA was extracted from each group of blood samples and the Agilent 2100 Bioanalyzer was used to assess RNA integrity shown in the top and bottom graphs separately (*n* = 3). (**B**) Genomic positions of circRNAs. Genomic profiles with commonly encountered circRNAs being shown in venous blood of C-Buffalo and P-Buffalo are displayed in the left and right panels, separately. (**C**) The GC content of circRNAs and mRNAs. Total GC amount in circRNAs and mRNAs for expression in C-Buffalo and P-Buffalo is displayed in the left and right panels.

Blood was collected from the jugular vein of the buffalo on the 25th day after insemination and later diagnosed by ultrasound pregnancy diagnostic techniques. The sequencing of venous blood collected from control buffaloes (C-Buffalo) and pregnant buffaloes (P-Buffalo) on day 25 of insemination was performed separately (Figure 1A). We defined particular circRNAs expressed in the blood of pregnant buffaloes by comparing circRNA transcripts from blood samples of C-Buffalo and P-Buffalo (Figure 1A). For verifying the reliability of the sequencing data, we gathered three samples per group with the association factor of 0.693~1 between biological replicates, indicating that the sequencing data were reliable. A total of 900,000 valid reads were acquired through the removal of aptamers and low-quality sequences, and mapping them to the buffalo reference genome (Supplementary Table S2). We ascertained a total of 57,308 circRNAs from 8612 host genes. Most host genes generated multiple different circRNAs, while a minority of host genes produced a single circRNA (27.26% in host genes). Analysis of the distribution of circRNAs in the genome showed that the majority the circRNAs were from exonic (83.82% in C-Buffalo and 83.19% in P-Buffalo) and intronic regions (12.29% in C-Buffalo, 12.82% in P-Buffalo), and a small proportion of circRNA was from intergenic regions (3.89% in C-Buffalo, 3.99% in P-Buffalo) (Figure 1B). The GC content of circRNAs averaged around 45% in C-Buffalo and P-Buffalo, similar to the 50% GC content of buffalo linear mRNA molecules (Figure 1C). Therefore, we separately identified and characterized lots of circRNAs expressed in the blood of C-Buffalo and P-Buffalo.

### 3.2. Determination of DECs in Blood between C-Buffalo and P-Buffalo

**Figure 2:**
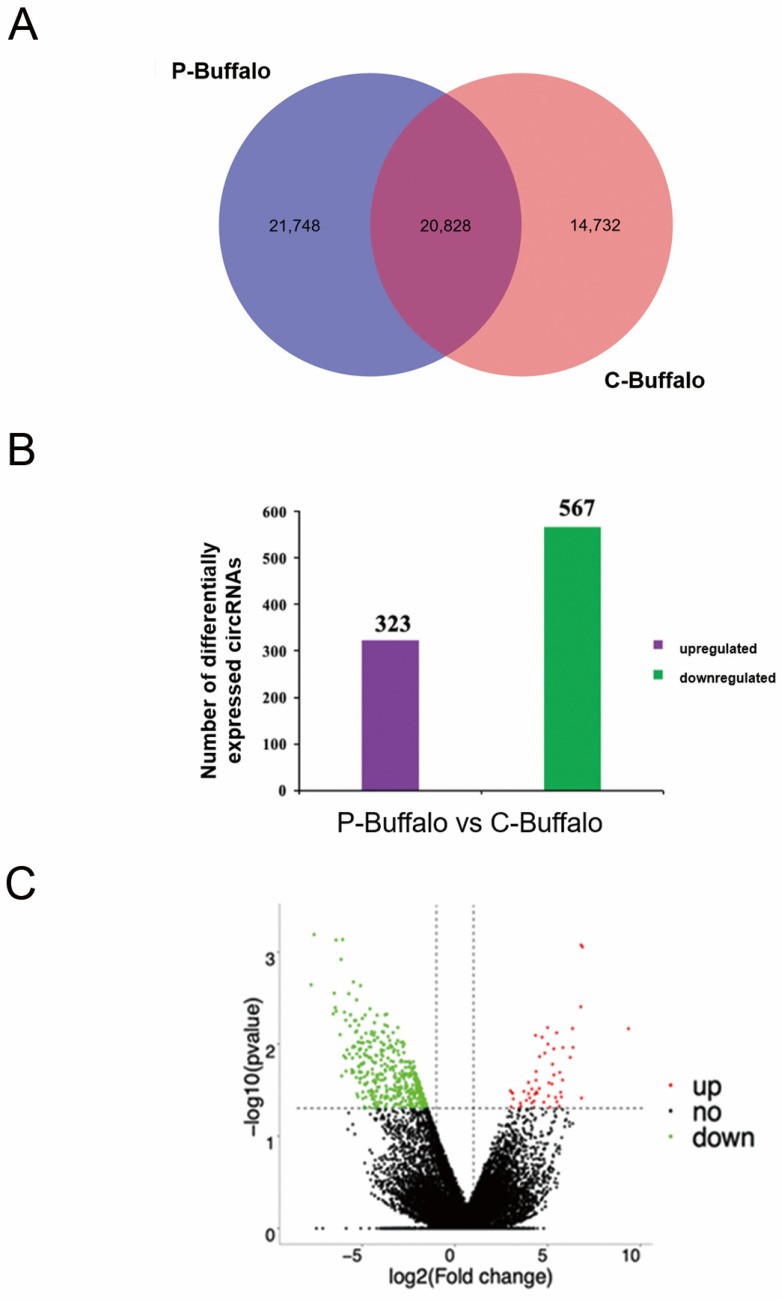
Identification of DECs in blood between C-Buffalo and P-Buffalo. (**A**) Venn diagram of circRNAs in P-Buffalo and C-Buffalo. Blood from C-Buffalo and P-Buffalo on day 25 after AI was subjected to RNA sequencing. The expression levels of circRNAs in the blood of buffaloes were analyzed by dichotomous statistical tests. Overlapping circles indicate circRNA, which was common in the blood of both groups of buffaloes. Circles that do not overlap indicate that circRNA is particular to (pink) non-pregnant buffalo and (blue) post-pregnant buffalo. (**B**) Amount of DECs in P-Buffalo and C-Buffalo. Adjusted results for *p* < 0.05 and |log2-fold change| ≥ 1 were considered statistically significant. Purple bar denotes up-regulated circRNAs; green indicates down-regulated circRNAs. (**C**) Volcano plot showing differences in circRNAs between P-Buffalo and C-Buffalo. Volcano map depicting differential expression between C-Buffalo and P-Buffalo. Differential expression of genes. Red shows up-regulated DECs, green depicts down-regulated DECs, and black diamond has no significant difference.

**Figure 3:**
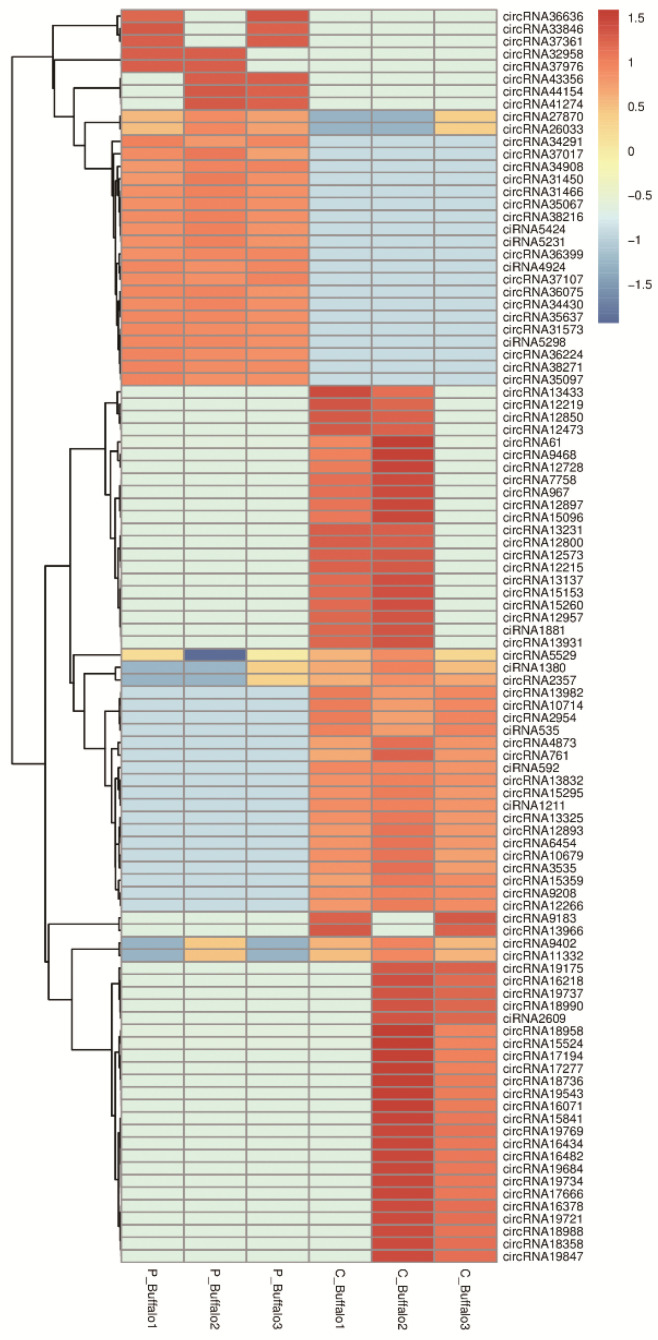
Heatmap of DECs in blood between C-Buffalo and P-Buffalo. Heatmap indicating the expression profiles of DECs in P-Buffalo and C-Buffalo. Red areas indicate up-regulated circRNAs whereas blue areas show down-regulated circRNAs. Heatmap color bars stand for expressed levels, with lightest blue representing −1.5 log2 fold change and darkest red representing 1.5 log2 fold change.

To assess the dynamics of circRNA expression in buffalo blood before and after pregnancy, we quantified the effective reads of RNA sequencing data in buffalo blood in C-Buffalo and P-Buffalo. Among the 57,308 circRNAs detected in the venous blood of both groups of cows, 20,828 circRNAs were co-expressed in the venous blood of both groups, with 21,748 and 14,732 being specifically expressed in P-Buffalo and C-Buffalo, respectively. (Figure 2A, Supplementary Table S3). In a comparison of buffalo blood in C-Buffalo and P-Buffalo, 8612 host genes produced 890 DECs, including 323 down-regulated and 567 up-regulated. Among them, there were 97 ciRNAs and 793 circRNAs (Figure 2B, Supplementary Table S2). More visualization of the differential genes between C-Buffalo and P-Buffalo was performed by venous blood sequencing, with screening conditions of *p*-adjust < 0.05 and |log2 fold change| ≥ 1 (Figure 2C). Next, we performed a hierarchical clustering analysis of the 100 circRNAs with the greatest differences in expression in the C-Buffalo and P-Buffalo. As the heatmap shows, the same phase of sample was aggregated together and the circRNA expression levels changed dynamically in C-Buffalo and P-Buffalo (Figure 3). Thus, we identified specific dynamic expression changes in thousands of circRNAs during gestation in Jianghuai buffaloes.

### 3.3. Functional Profiling for Host Genes of DECs between C-Buffalo and P-Buffalo

**Figure 4:**
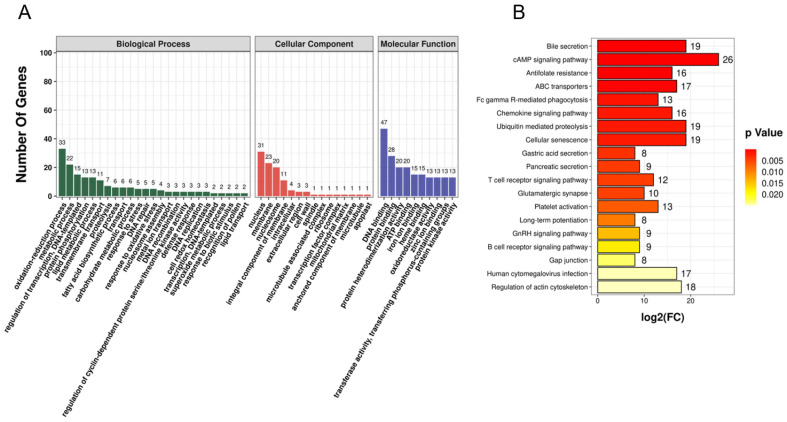
The GO and KEGG analysis of host genes of DECs between P-Buffalo and C-Buffalo. (**A**) An examination of the top enriched terms of the DEC host genes found in P-Buffalo and C-Buffalo was performed by GO analysis. the DEC host genes were divided into three classes (blue bars: biological processes, green bars: cellular components, orange bars: molecular functions). (**B**) Enrichment profiling of the top KEGG pathway for host genes of DECs discovered in P-Buffalo and C-Buffalo.

In existing studies finding that circRNA function might be correlated with the known function of host genes, we analyzed GO and KEGG pathways of host genes that produce DECs to anticipate their potential function in C-Buffalo and P-Buffalo. Depending on the GO database, there are three main categories of host genes that produce DECs in venous blood (biological process, cellular component, and molecular function). A total of 2687 GO terms were enriched for the 8612 host genes producing DECs in venous blood, of which 132 GO terms were significantly enclosed for 3 GO functions or functions to be determined (*p* < 0.05). Of the 132 GO terms that were significantly enriched, 83 were under “biological process”, 22 were under “cellular component” and 27 were under “molecular function”. The top 25 biological processes, 15 cellular components, and 10 molecular functions list the host genes involved in each GO term (Figure 4A, Supplementary Table S4), such as negative regulation of transcription by RNA polymerase II (10 genes, e.g., *RCOR3*, *NFKB1*, and *SMAD3*), cytoplasm (5 genes, namely *ARHGAP6*, *TAB3*, *USP9X*, and *PPP1R9A*), regulation of transcription by RNA polymerase II (9 genes, e.g., *RCOR3*, *LOC102391826*, *SMAD3*, *BRWD1*, and *TFDP2*), and protein binding (4 genes, e.g.,*VPS35L*, *RCOR3*, *DNM3*, *SETD5*, *TTC39B*) (Supplementary Table S4) (*p*-adjust < 0.05).

### 3.4. Predicting Potential Sponging miRNA Targets for circRNAs Expressing in P-Buffalo and C-Buffalo

In addition, KEGG analysis of host genes for venous blood production of DECs revealed 32 significant pathways (Figure 4B, Supplementary Table S5), including the cAMP signaling pathway (26 genes, Antifolate resistance (16 genes), GnRH signaling pathway (9 genes), C-type lectin receptor signaling pathway (10 genes), hippo signaling (14 genes), Platelet activation (13 genes), Chemokine signaling pathway (16 genes), and B cell receptor signaling pathway (9 genes) (*p*-adjust < 0.05).

In general, the above pathways produced in differential gene enrichment in C-Buffalo and P-Buffalo venous blood were mainly associated with cell gap junctions and cell differentiation. These results suggest that there may be a strong association between these DECs and the establishment of pregnancy-related signaling pathways.

**Figure 5:**
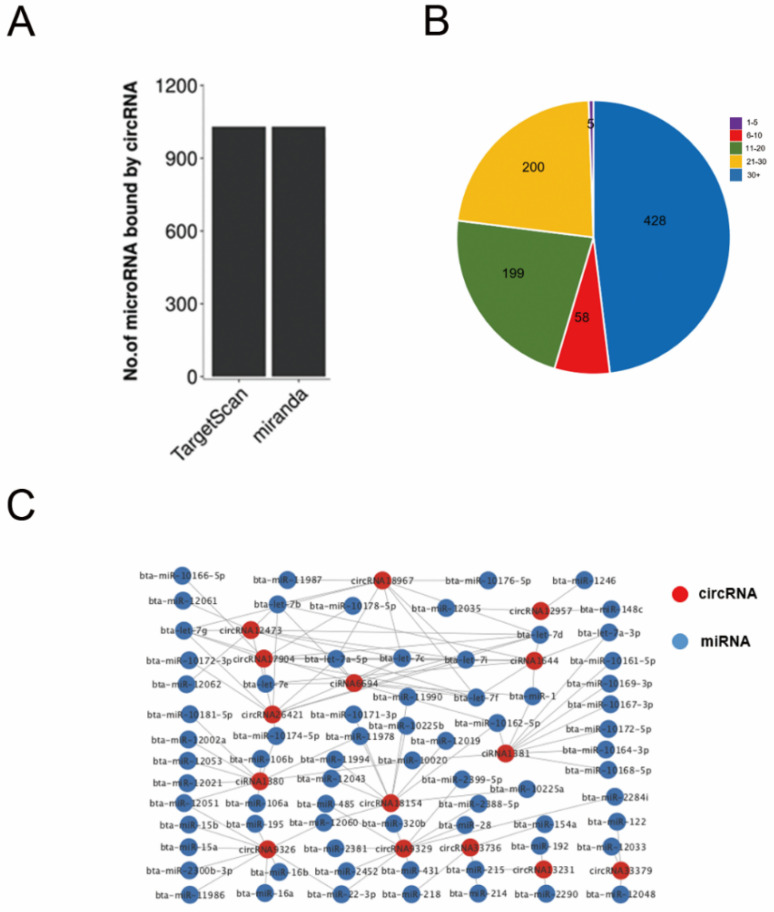
Analysis of interaction between DECs and miRNAs between P-Buffalo and C-Buffalo. (**A**) Analysis of the circRNAs for miRNAs with TargetScan and Miranda. (**B**) Identification of the percentages of circRNAs with different numbers of miRNA targets. (**C**) Analysis of the predicted target miRNAs for selected DECs identified in c and P. The circRNAs selected were picked from the top DECs. Red circles indicate circRNAs and blue circles show miRNAs.

CircRNAs function as miRNA sponges that indirectly regulate the expression in target genes by adsorbing miRNAs. This process is an important form in post-transcriptional regulation, which predicts miRNA binding sites on circRNAs is beneficial to reveal the potential roles of circRNAs. Using predictions from software such as TargetScan and Miranda, we found a total of 1030 miRNA binding sites on 890 DECs (Figure 5A, Supplementary Table S6). Each circRNA can bind to one or several miRNA targets. Therefore, in our study, we further analyzed circRNAs containing various numbers of miRNA targets. The vast majority of circRNAs had at least two miRNA binding sites, with the largest proportion of circRNAs containing more than 30 miRNA targets. (Figure 5B). Functional analysis of the source genes predicted that circ*SMAD3*, circ*BIRC6*, ciSOX6, circ*NEDD4*, circ*SOX6* (down-regulated), and circ*LOC102411048*, circ*TRIP12*, circ*FOXP1* (up-regulated) may be associated with pregnancy recognition or embryo establishment. To more clearly demonstrate the predicted relationships between circRNAs and miRNAs, we mapped circRNA-miRNA interaction networks using software such as Cytoscape (Figure 5C). These data suggest that the majority of DEGs in C-Buffalo and P-Buffalo blood do contain multiple miRNA targets.

### 3.5. Validation of DECs in Blood between C-Buffalo and P-Buffalo

**Figure 6:**
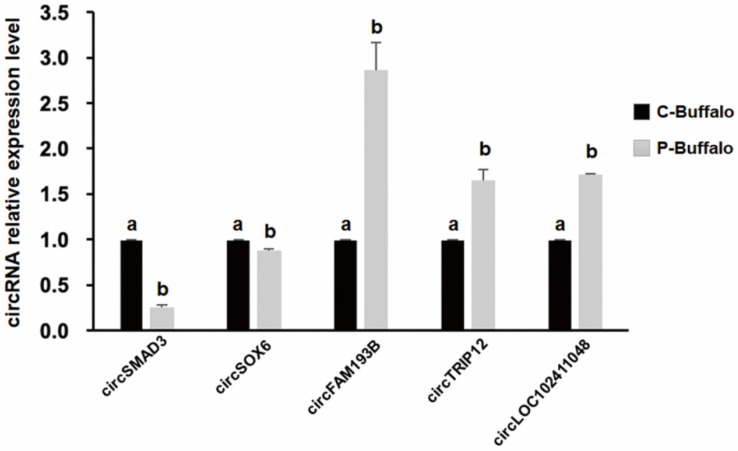
Validation of the selected DECs identified between P-Buffalo and C-Buffalo. The selected circRNAs were chosen from the DECs of venous blood of C-Buffalo and P-Buffalo. The relative abundance of circRNAs C-Buffalo and P-Buffalo was quantified by qPCR. Normalization of the data with the endogenous housekeeping gene *GAPDH* was performed, and the number of values for C-Buffalo was set to 1. Different letters on the bars indicate significant differences (*p* < 0.05). Data are presented as mean ± S.E.M. Statistical analysis was done by t-student test.

In order to confirm the authenticity of circRNA sequencing data, five circRNA expression levels (two down-regulated in P-Buffalo, three up-regulated in P-Buffalo), namely circ*SMAD3*, circ*SOX3*, circ*FAM193B*, circ*TRIP12*, circ*LOC102411048* were analyzed by qPCR (Supplementary Table S1). The expression pattern of this selected circRNA during pre- and post-pregnancy in buffalo is highly comparable to the results derived from circRNA sequencing data (Figure 6). Thus, these data confirmed the reliability of the circRNA sequencing data.

## 4. Discussion

As a novel non-coding RNA, the high stability and species conservation of circRNA in mammals have become a new hot spot for research in recent years. Much research has reported that circRNAs are extensively present in humans [31], mice [32], pigs [33], sheep [34], cattle [35], and other mammals. The expression profiles of circRNAs in different tissues of cattle are well studied, but their expression and functions in buffalo pregnancy remain to be investigated. In this study, we extracted total RNA from jugular vein blood of C-Buffalo and P-Buffalo and identified a total of 57,308 circRNAs in six circRNA libraries constructed, which is close to the number of circRNAs identified in human blood exosomes by Li [22], and the result implies that circRNA expression may be highly conserved in buffalo and human blood [17,36]. It has been reported that circRNAs are highly enriched in human peripheral blood and circRNAs in human blood are trending towards clinical diagnosis as molecular markers for various diseases [36]. Therefore, the results of this study not only complement the study of circRNAs in buffalo [37] but also provide a theoretical basis for the use of circRNAs as a clinical molecular marker in the blood of Jianghuai buffaloes. The GC content of circRNAs in Jianghuai buffalo blood was around 45%, which was more similar to the GC content of circRNAs identified by Cao [33] in porcine cumulus cells and oocytes. This also suggests that the stability of circRNAs is conserved among species, which may be related to the unique loop structure of circRNAs. Eight hundred and ninety DECs out of the 57,308 circRNAs were found, most of which were ecRNAs, which is consistent with the findings of Zhang [37] in bovine mammary tissue. Progesterone plays an important part in gestational recognition as well as in the maintenance of pregnancy. During early pregnancy, the corpus lutetium synthesizes and secretes progesterone, and progesterone levels in the blood are significantly elevated. Jia [38] found that knocking down circ*EGFR* in mouse ovarian granulosa cells significantly promoted progesterone synthesis. In our study, 323 circRNAs were found to be up-regulated and 567 circRNAs were down-regulated in the pregnant group compared to the non-pregnant group, including circ*EGFR*. Therefore, we speculate that some circRNAs down-regulated in the blood of Jianghuai buffalo may be involved in the regulation of progesterone synthesis and secretion, and the specific molecular mechanisms need to be further investigated.

It has been demonstrated that circRNAs can cis-regulate the expression levels of their host genes by regulating RNA polymerase, and the knockdown of some circRNAs significantly inhibits the expression levels of their host genes; therefore, the underlying functions of circRNAs may be more closely related to the functions of their host genes [39]. We conducted GO and KEGG analyses on the parental genes of the DECs. The GO analysis revealed that these parental genes are primarily associated with negative regulation of RNA polymerase II transcription, cytoplasmic composition, and ATP binding, as well as positive regulation of the epidermal growth factor receptor (*EGFR*) [14]. Kyoto Encyclopaedia of Genes and Genomes analysis has shown that these parental genes are associated with the cell gap junction pathway. Gap junctions can play a multifaceted role by facilitating the diffusion of signaling molecules and acting as a direct channel for cell-to-cell communication [40]. One study found that the cell gap junction protein Cx43 maintains placental growth and plays a potential role during pregnancy in rats and rabbits [41]. Therefore, together with the above functional analysis, we speculate that these DECs may be closely associated with the establishment of pregnancy-related signaling pathways.

One of the functions of circRNAs is as miRNA sponges that indirectly regulate the expression of target genes through adsorption of miRNAs, and this process is an important post-transcriptional regulation of the miRNA binding sites that are predicted to help reveal the potential role of circRNAs. Using predictions from software such as TargetScan and Miranda, we found a total of 1030 miRNA binding sites on 890 DECs, and the results predicted that the DECs associated with pregnancy in Jianghuai buffalo are not in one-to-one correspondence with miRNAs, and most circRNAs contain more than 30 miRNA binding sites. In a study of fatty acid regulatory mechanisms in cow mammary epithelial cells, Chen [42] found that circ09863 further regulated fatty acid metabolic processes by competitive binding of miR-27a-3p to attenuate the inhibitory effect of miR-27a-3p on fatty acid synthase expression. Based on this, we hypothesized that the circRNA-miRNA-mRNA regulative network may also exist during gestational recognition and early embryonic development in Jianghuai buffalo, which provides an idea to study the specific regulatory mechanism of circRNA in ruminant embryonic development.

Previous studies have found that circRNA plays an important role in human reproductive medicine. Qian [43] reported highly up-regulated circRNAs at the onset of pre-eclampsia and hypothesized that circRNAs are involved in regulating the pathogenesis of pre-eclampsia. Some studies have found that changes in circRNA expression patterns are also observed in the endometrium of patients with recurrent miscarriages. Zhu [44] reported that circRNA-DURSA regulates the apoptosis of embryonic trophectoderm cells in recurrent spontaneous abortion via the miRNA-760-HIST1H2BE axis. This suggests that specific circRNAs are likely to be candidate biomarkers or molecular targets for unexplained recurrent spontaneous abortion. In addition, some studies have found that ciRS-7, as a sponge for miR-7, is expressed at a higher level in samples of ovarian tumors [45]. In summary, circRNAs have a potential impact not only on human reproductive diseases but also on the development of biomarkers for ovarian-associated cancers, as reported in previous studies.

In this study, tens of thousands of circRNAs were identified by high-throughput sequencing technology. Large numbers of DEGs were present in the blood of 25d pregnant cows and non-pregnant cows. These DEGs are not only associated with pregnancy-related pathways, but also closely linked to many diseases. Follow-up research can take advantage of the unique advantages of circRNAs to develop an efficient, accurate and minimally invasive technique for early pregnancy diagnosis. This suggests that the expression pattern of circRNAs in the blood can be measured to assess the continued study of biomarkers or biological targets associated with pregnancy disorders, and this will have great academic value and application in the future.

## 5. Conclusions

In conclusion, these results suggest that there is an abundance of circRNAs in the blood of Jianghuai buffaloes, thousands of which are expressed in a specific manner during gestation. Our data also suggest that these DECs are mainly enriched in important pathways related to germ-line recognition and intercellular communication. Further validation of the function of highly differential DECs in gestational recognition is needed.

## Data Availability

The data presented in the study are deposited in the GEO repository, accession number (GSE208385).

## Supplementary Materials

The following supporting information can be downloaded at: https://www.mdpi.com/article/10.3390/ani12202748/s1, Supplementary Table S1. Bubalus bubalis-specific primer sequences used in this study. Supplementary Table S2. List of all circRNAs expressed in buffaloes. Supplementary Table S3. List of circRNAs in C-Buffalo and P-Buffalo. Supplementary Table S4. GO analysis of host genes producing DECs in buffaloes. Supplementary Table S5. KEGG analysis of host genes producing DECs in buffaloes. Supplementary Table S6. circRNA-miRNA interaction analysis.
