# Supplementary material for: Expression Profiling of Circular RNAs in Early Pregnant Jianghuai Buffaloes
Source: Animals (Basel). 2022 Oct 13;12(20):2748. doi: 10.3390/ani12202748 (PMC9597768; doi:10.3390/ani12202748)
Supplement: Supplementary file 1 [file animals-12-02748-s001.zip › Table S1.pdf]

Table S1. ***Bubalus bubalis*** **-specific primer sequences used in this study**

Gene symbol	Primer sequences (5'-3')
circSMAD3	F: CCACAGCATGGATGCAGGTTC<br>R: AGGCGGCAGTAGATGACATGAG
circSOX6	F: CGTTTGGGCAGGAGTTTGGAC<br>R: TGGAGCTGTAAAGGGCTGAGT
circFAM193B	F: CGTAGCAGAAACAGTCGCCA<br>R: ACATCGTTAGACAGCAGGTTGA
circTRIP12	F: GAGAAGATGGCCGACCCTGA<br>R: TGGACATTGGCACCTCTCTCTT
circLOC102411048	F: ACGATGTGAACAGGTTTGACAAG<br>R: TCAGTACAGCAGTGTAGCCA
GAPDH	F: GGGTGTGAACCACGAGAAGT<br>R: CGGTGGTCATAAGTCCCTCC
**F: forward,**<br>**R: reverse**	
